# Self-organizing maps with variable neighborhoods facilitate learning of chromatin accessibility signal shapes associated with regulatory elements

**DOI:** 10.1186/s12859-021-03976-1

**Published:** 2021-01-30

**Authors:** Tara Eicher, Jany Chan, Han Luu, Raghu Machiraju, Ewy A. Mathé

**Affiliations:** 1 Department of Biomedical Informatics, The Ohio State University College of Medicine, 370 W. 9th Avenue, Columbus, OH 43210 USA; 2 Department of Computer Science and Engineering, The Ohio State University College of Engineering, 2015 Neil Avenue, Columbus, OH 43210 USA; 3 Division of Preclinical Innovation, National Center for Advancing Translational Sciences, National Institute of Health, 9800 Medical Center Dr., Rockville, MD 20892 USA; 4 Department of Pathology, The Ohio State University College of Medicine, 1645 Neil Ave, Columbus, OH 43210 USA; 5 Translational Data Analytics Institute, The Ohio State University, 1760 Neil Ave., Columbus, OH 43210 USA

**Keywords:** Chromatin accessibility, Self-organizing maps, Regulatory elements, DNase-seq, ATAC-seq, Promoters, Enhancers, Chromatin state assignment, RPKM signal shape, Machine learning

## Abstract

**Background:**

Assigning chromatin states genome-wide (e.g. promoters, enhancers, etc.) is commonly performed to improve functional interpretation of these states. However, computational methods to assign chromatin state suffer from the following drawbacks: they typically require data from multiple assays, which may not be practically feasible to obtain, and they depend on peak calling algorithms, which require careful parameterization and often exclude the majority of the genome. To address these drawbacks, we propose a novel learning technique built upon the Self-Organizing Map (SOM), Self-Organizing Map with Variable Neighborhoods (SOM-VN), to learn a set of representative shapes from a single, genome-wide, chromatin accessibility dataset to associate with a chromatin state assignment in which a particular RE is prevalent. These shapes can then be used to assign chromatin state using our workflow.

**Results:**

We validate the performance of the SOM-VN workflow on 14 different samples of varying quality, namely one assay each of A549 and GM12878 cell lines and two each of H1 and HeLa cell lines, primary B-cells, and brain, heart, and stomach tissue. We show that SOM-VN learns shapes that are (1) non-random, (2) associated with known chromatin states, (3) generalizable across sets of chromosomes, and (4) associated with magnitude and multimodality. We compare the accuracy of SOM-VN chromatin states against the Clustering Aggregation Tool (CAGT), an unsupervised method that learns chromatin accessibility signal shapes but does not associate these shapes with REs, and we show that overall precision and recall is increased when learning shapes using SOM-VN as compared to CAGT. We further compare enhancer state assignments from SOM-VN in signals above a set threshold to enhancer state assignments from Predicting Enhancers from ATAC-seq Data (PEAS), a deep learning method that assigns enhancer chromatin states to peaks. We show that the precision-recall area under the curve for the assignment of enhancer states is comparable to PEAS.

**Conclusions:**

Our work shows that the SOM-VN workflow can learn relationships between REs and chromatin accessibility signal shape, which is an important step toward the goal of assigning and comparing enhancer state across multiple experiments and phenotypic states.

## Background

Regulatory elements (REs) denote regions of chromatin that promote, enhance, repress, or insulate transcription [1, 2]. Genetic variants or epigenetic modifications in REs are associated with many diseases, including migraines [3–5], cancer [6–8], coronary artery aneurism [9, 10], and spinal muscular atrophy [11, 12]. However, globally associating regions of the human genome with REs is experimentally expensive in that it typically requires multiple assays.

The location of REs can be measured genome-wide using chromatin-accessibility-based methods, such as FAIRE-seq [13], DNase-Seq [14], ATAC-seq [15], NicE-seq [16], MNase-seq [17], and NOMe-seq [18], as reviewed in [19]. In addition, machine learning methods have been developed to assign chromatin state, including Spectacle [20], CoSBI [21], Segway [22], and ChromHMM [23]. However, assigning enhancer, promoter, or other chromatin states requires evaluation of histone modifications or transcription factors [2, 24, 25]. This is typically done using multiple ChIP-seq assays, which require 1–20 million cells per assay [26] or specialized protocols and antibodies [27].

Thus, new computational methods are needed to assign chromatin state at the genome scale from a single chromatin accessibility experiment. Ideally, these computational methods should include signal shape rather than peak location alone. Traditional peak caller performance in DNase-seq assays is heavily dependent on method and parameter choice [28] and may therefore miss variations in chromatin accessibility signal that are relevant to the task of assigning chromatin state. Signal shape, on the other hand, captures the richness of these variations and has been leveraged successfully in related tasks, such as identifying peaks missed by traditional peak callers [29, 30], clustering regions in ChIP-seq assays [21], and characterizing transcriptional regulation and gene expression [31].

Currently, only 2 methods, Predicting Enhancers from ATAC-Seq Data (PEAS) and the Clustering Aggregation Tool (CAGT), can assign chromatin state genome-wide using chromatin accessibility data alone [32]. PEAS uses peak shape characteristics and optional genomic features including GC content and sequence motif to assign chromatin state to peaks after promoters have been removed. While PEAS achieves high accuracy, it (1) only assigns chromatin state within peaks, not the entire genome; (2) is limited to differentiating enhancer states from other REs after promoters have been removed; and (3) uses deep learning and thus has limited interpretability in terms of the function by which shape and genomic features predict chromatin state assignment. CAGT uses a combination of *k*-means clustering and hierarchical clustering to associate chromatin accessibility signal shape (i.e. variation of signal intensities across a region) with other epigenetic factors (e.g. transcription factor binding sites, nucleosome positioning, and sequence content), which yields more interpretable results. Unlike PEAS, CAGT makes use of signal shape across the genome rather than peaks alone. However, CAGT is normally used to study transcription start sites (TSS) rather than whole chromosomes and has not been applied to chromatin state assignment. This makes applicability to genome-scale assignments unclear, as only 5% of chromatin accessibility peaks are located within 2.5 kb of TSS [33].

**Fig. 1:**
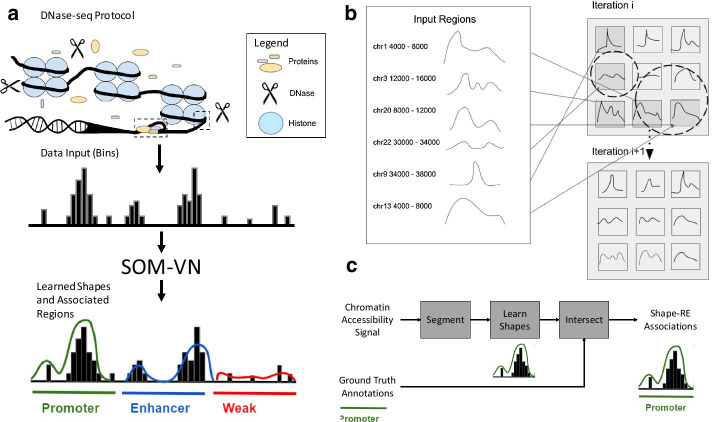
The SOM-VN workflow learns chromatin accessibility signal shapes and associates them with REs. **a** SOM-VN learns signal shapes from input DNase-seq signal which are then assigned to RE-associated chromatin states (e.g. promoter, enhancer, weak). **b** To learn shapes on each chromosome, SOM-VN uses an iterative training process which operates on a grid of nodes, where each node comprises one shape. Dotted circles represent the neighborhood of each node in an iteration. **c** Normalized DNase-seq signal segmented into regions is used as input to the SOM-VN training process. The learned shapes are then associated with ChromHMM annotations from Roadmap Epigenomics

Here, we present an improvement on the Self-Organizing Map (SOM), which we call SOM with Variable Neighborhoods (SOM-VN), by incorporating a signal-dependent calculation of neighborhood sizes when learning chromatin accessibility signal shapes from regions segmented across entire chromosomes. A SOM is a single-layer neural network where each node and weight corresponds to a shape from a chromosomal region and its signal intensity, respectively [34]. In our implementation, we enabled variation of a key parameter, neighborhood size, according to criteria based on signal intensity within a learned shape (Fig. 1). This enhancement allowed us to address imbalances in chromatin accessibility data where the majority of the genome is largely inaccessible (or below the floor) [35]. We then associated the set of shapes learned with ChromHMM annotations from the Roadmap Epigenomics Project, which incorporate experimental annotations such as CpG islands, GENCODE transcription start sites, and transcription factor binding sites [36].

## Results

We tested SOM-VN using 14 publicly available DNase-seq assays representing diverse cell types, data quality, and read count (Supplementary Table 1 in Additional file 1) from ENCODE. These included 4 cell lines, 1 primary cell culture, and 3 tissue samples. We determined the quality of each sample using the Signal Portion of Tags (SPOT) score defined by ENCODE, which approximates signal to noise ratio using the percentage of reads falling into peaks. We obtained samples with SPOT scores below and above the recommended threshold set by ENCODE (0.4) [37] for all cell types except A549 and GM12878, for which samples above the recommended SPOT scores were unavailable. With the exception of the GM12878 and H1 cell types, all samples with low SPOT scores also had read depths below the recommended threshold set by ENCODE (50 million) [37]. For the remainder of this manuscript, we denote high SPOT samples with (H) and low spot samples with (L).

We concatenated reads for isogenic replicates in GM12878 and A549 cell types, and we concatenated reads for biological replicates in brain tissue (H). The percentage of peaks passing an Irreproducible Discovery Rate cutoff of 0.05 across replicates was greater than 50% in all samples. Likewise, when we learned shapes on each replicate separately using SOM-VN and used these to assign chromatin state, we found between 20 and 80% overlap for all REs in all cell types (Supplementary Table 2 in Additional file 1).

### Validation of model

We tested SOM-VN against 2 null models. To evaluate the extent to which shapes were representative of the variations in signal across regions in a chromosome, we computed the cross-correlation between regions and their matching shapes and compared these against the cross-correlation between regions and matching shapes learned using permuted signal. To evaluate the veracity of associations between shapes and chromatin states as represented by REs, we permuted the ChromHMM annotation file for each cell type, associated shapes with REs using the permuted annotations, and validated that the count and variety of associations learned were lower than using the original annotation file.

**Fig. 2:**
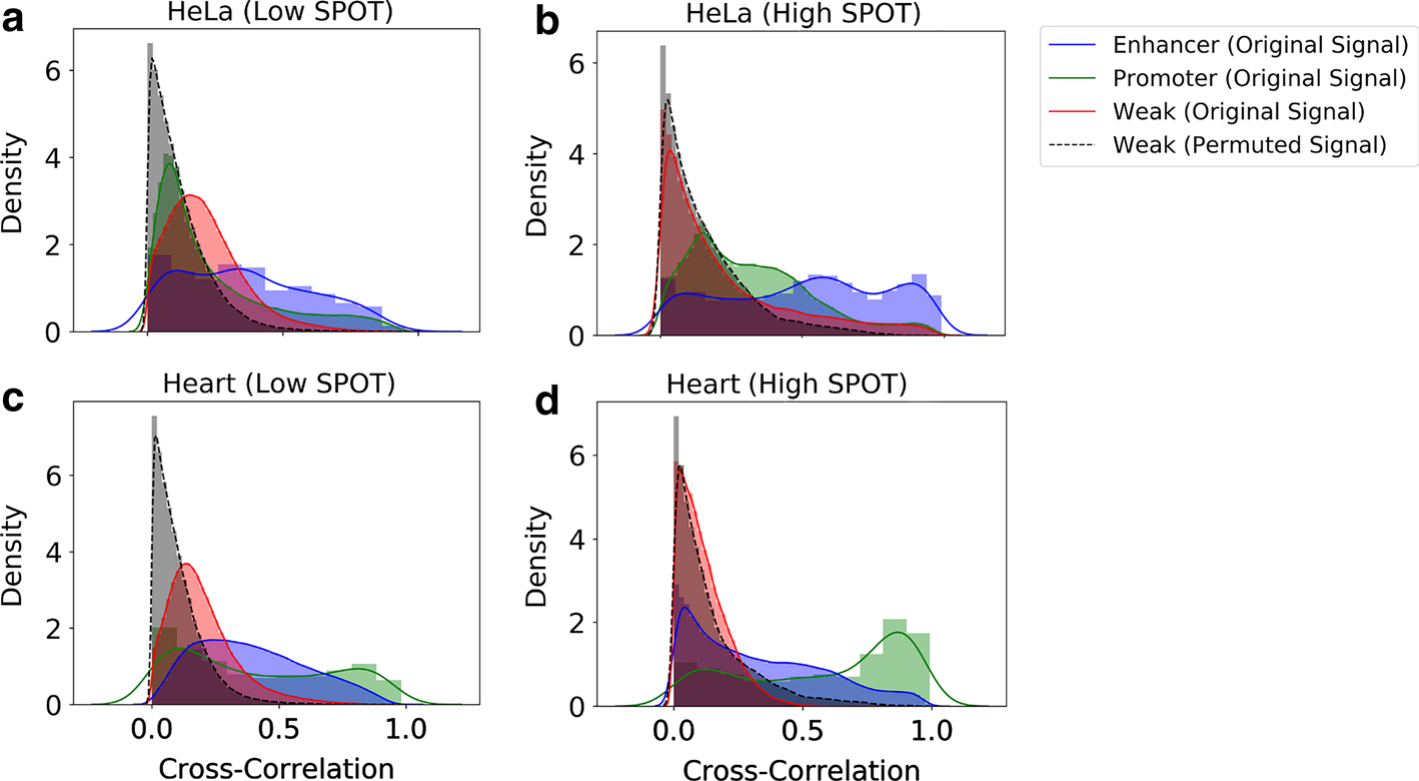
Regions exhibited higher cross-correlation with their matching shapes than with randomly learned shapes. The density plots indicate the distribution of cross-correlations between regions and their matching shapes for all regions, and the color denotes the RE annotation (red—weak, blue—enhancer, green—promoter). Statistically higher cross-correlations were found between shapes learned using the original, unpermuted signal and their matching regions than between shapes learned using permuted signal and their matching regions. In all samples, the permuted shapes were associated with only weak or unknown REs

We used the Wilcoxon Rank-Sum Test to validate that the difference between cross-correlation distributions for permuted and unpermuted signal differed significantly. For all REs and all samples, the difference was statistically significant at a Bonferroni-adjusted cutoff of *p* = 0.05 (Supplementary Table 3 in Additional file 1). Distributions are shown in Fig. 2 and in Supplementary Fig. 1 in Additional file 2.

This result suggests that the shapes learned are more reflective of signal variations in the input than are randomized shapes. We note that, in a minority of samples, shapes that were associated with enhancer annotations were not uncovered: namely, H1 (L, H) and GM12878.

Notably, all shapes learned using permuted signal were associated with weak REs or had no association, suggesting that the shapes associated with enhancers and promoters are only recoverable in the original signal. In brain tissue (H), the distribution of cross-correlations skews higher than in other samples, which may reflect lower signal-to-noise ratio in brain tissue (H) and the high read counts of the replicates in this sample (Supplementary Table 4 in Additional file 1).

In the second null model, the permuted annotation model, only a single enhancer-associated shape was found in 1 sample (i.e. A549), and no promoter-associated shapes were found in any sample. In contrast, the associations between unpermuted REs and shape included 60 promoter-associated shapes and 3 enhancer-associated shapes. All other associations in the permuted annotation model were with weak REs. This result is expected because of the imbalance in RE type coverage, as weak REs cover a higher percentage of the genome than promoter or enhancer REs (Supplementary Table 4 in Additional file 1). We conclude that SOM-VN identifies non-arbitrary linkages between shape and annotation using chromatin accessibility experiments alone.

### Peak coverage of SOM-VN chromatin states

Because SOM-VN learns shapes without depending on peak callers, we evaluated whether there was any relationship between peaks and chromatin state assignments made by SOM-VN. We computed the percentage of promoter, enhancer, and weak assigned regions overlapping peaks (Supplementary Table 5 in Additional file 1). We found that between 86 and 100% of promoter-assigned regions overlapped peaks, compared to between 36 and 100% of enhancer-assigned regions and between 3 and 60% of weak-assigned regions. This result supports the association between open chromatin and promoters, and to a lesser extent, open chromatin and enhancers.

### Robustness of model across chromosomes

**Fig. 3:**
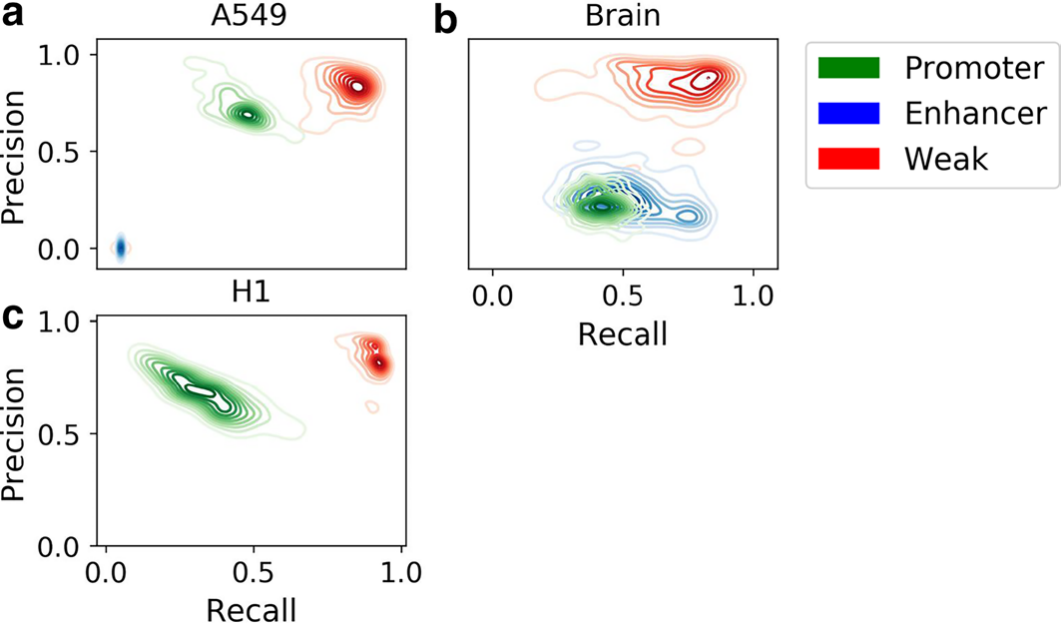
The selection of chromosomes used for learning had little impact on precision and recall. Precision and recall were evaluated across 100 cross-chromosome runs, in which shapes were learned on 11 randomly-selected autosomes and used to assign RE annotations to the remaining 11 autosomes. The tightness of the contours represents the spread of precision and recall values for each RE type across all runs, and the colors of the contours correspond to RE (red—weak, blue—enhancer, green—promoter). As exhibited by the low spread of precision and recall indicated by the contours, the choice of chromosomes did not significantly impact precision and recall, indicating generalizability of the workflow across chromosome subsets in **a** A549, **b** brain tissue, and **c** H1

To evaluate whether SOM-VN yielded generalizable results across chromosomes, we calculated the precision and recall values obtained when training on 100 randomized subsets of 11 autosomes and testing on the remaining autosomes for the A549 sample, the H1 (L) sample, and the brain tissue (H) sample. Within cell types, precision and recall calculated in the test autosomes did not vary greatly for different sets of autosomes chosen for training (Fig. 3). All RE and cell types exhibited some consistency. Notably, chromatin state assignment performance was most consistent across iterations in H1 and A549 weak REs and in A549 and brain tissue promoters. Overall, brain tissue (H) had higher enhancer recall on average (0.53) than H1 (L) (0.01) and A549 (0.03) and lower promoter recall on average (0.28) than H1 (L) (0.67) and A549 (0.73), suggesting that chromatin state assignment performance exhibits some tradeoff between promoter and enhancer recall. In addition, precision values between 0.65 and 0.88 and recall values between 0.77 and 0.81 were found in weak REs for all samples. We note that weak RE states representing areas of heterochromatin could contribute to gene regulation [38].

### Characteristics of shapes

**Fig. 4:**
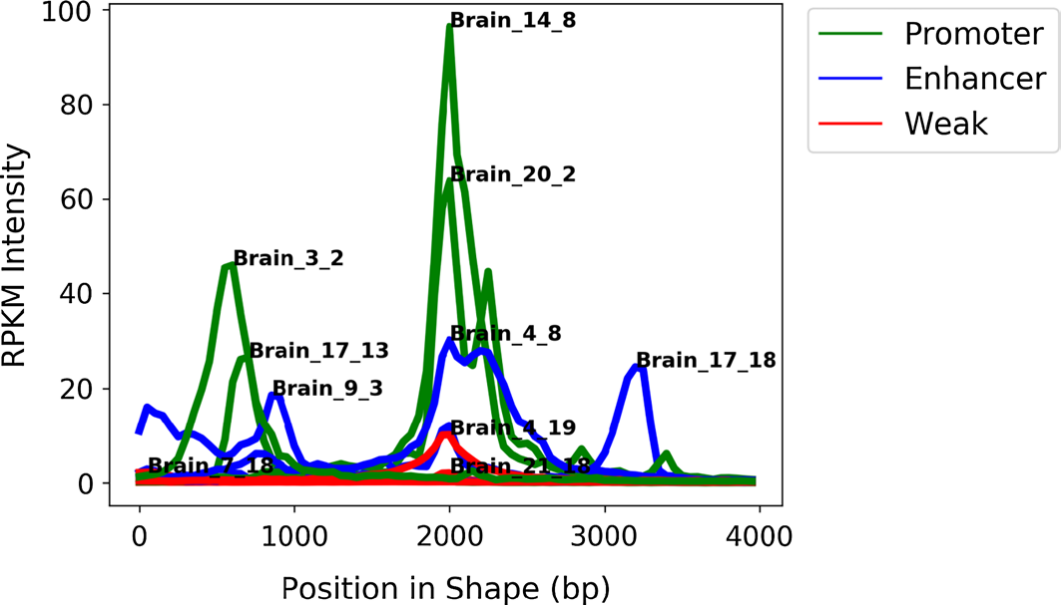
Example shapes learned on brain tissue exhibited differences in magnitude and multimodality by RE. In selected example shapes from brain tissue, differences in summit height and bimodality were apparent between promoter (green), enhancer (blue), and weak REs (red), where the name annotated near each shape’s summit is in Cell-type_chromosome_SOM-VN-node format

We visualized a subset of shapes learned by SOM-VN on all chromosomes from brain tissue (H) (Fig. 4). A notable result was that promoter-associated shapes had the highest summits. This result was consistent with existing knowledge that chromatin is more accessible in regions proximal to TSS than in distal regions [33]. Intriguingly, some enhancer-associated shapes shown in Fig. 4 were bimodal or multimodal, suggesting that these characteristics could be used to identify enhancer regions.

To evaluate whether these patterns were observable in other cell types, we computed the range of maxima for each RE and cell type, and we visually determined the percentage which were bimodal or multimodal. We found that, in general, promoter-associated shape maxima were higher than enhancer-associated shape maxima, which were higher than weak-associated shape maxima; however, some overlap existed between the groups (Supplementary Table 6 in Additional file 1). Additionally, we found that the percentage of enhancer-associated shapes that were multimodal was larger than the percentage of promoter-associated shapes that were multimodal in high SPOT data, which provided additional support for the relationships between multimodality and enhancers (Supplementary Table 6 in Additional file 1). We note that additional characteristics of shape could also be explored in future studies.

### Generalization of shapes across cell type

Because the shapes learned using SOM-VN were applicable across chromosomes and exhibited visual characteristics, we next tested whether these shapes were generalizable across cell type. We learned shapes using SOM-VN on chromosomes of one sample and used them to assign chromatin state in another sample. To evaluate the generalization across cell type, we evaluated the following pairs: A549 to brain tissue (H) and H1 (L), brain tissue (H) to A549 and H1 (L), and H1 (L) to A549 and brain tissue (H). We indicate the mean values across all chromosomes in Table 1.

**Table 1:** Generalization of shapes across cell type was cell type dependent

Training sample	Testing sample	Promoter		Enhancer		Weak	
		Precision	Recall	Precision	Recall	Precision	Recall
*A549*	*A549*	0.39	0.84	0.31	0.06	0.88	0.76
	*Brain (H)*	0.16	0.79	0.38	0.05	0.89	0.62
	*H1 (L)*	0.19	0.91	0.28	0.10	0.90	0.26
*Brain (H)*	*Brain (H)*	0.45	0.26	0.33	0.34	0.75	0.80
	*A549*	0.72	0.25	0.24	0.30	0.66	0.83
	*H1 (L)*	0.74	0.13	0.26	0.33	0.80	0.86
*H1 (L)*	*H1 (L)*	0.37	0.72	N/A^a^	N/A	0.81	0.90
	*A549*	0.47	0.69	N/A	N/A	0.66	0.77
	*Brain (H)*	0.20	0.62	N/A	N/A	0.74	0.75

^a^N/A values indicate that no shapes were learned for the corresponding RE in the corresponding sample

Specific types of shapes learned using SOM-VN were generalizable between 2 cell types. For example, enhancer-associated shapes learned on brain tissue were generalizable to A549. Similarly, when shapes learned on H1 were applied to brain tissue, weak RE precision and recall did not differ considerably from when the same shapes were applied back to H1 itself. Interestingly, even though no enhancer-associated shapes were learned on H1, enhancer-associated shapes learned on brain tissue were generalizable to H1. These results indicate that enhancer and weak RE associated shapes learned using SOM-VN can generalize across some cell types.

### Comparison to baselines

While only SOM-VN is designed to learn chromatin accessibility signal shapes across whole chromosomes to assign chromatin to RE-associated states, CAGT was designed for a similar task. We therefore compared the performance of SOM-VN to this method. To compare against CAGT, we learned shapes using the default parameters for CAGT and then associated them with REs (see Methods). For each sample, we evaluated the precision and recall of chromatin state assignment for each chromosome using shapes learned on all chromosomes (Table 2).

**Table 2:** SOM-VN improved precision and recall over CAGT and magnitude-based models

Sample	Method	Promoter		Enhancer		Weak	
		Precision	Recall	Precision	Recall	Precision	Recall
*A549*	CAGT	0.14	0.53	0.24	0.10	0.77	0.58
	Magnitude	0.15	1.00	N/A	N/A	0.05	0.00
	SOM-VN	0.19	0.86	0.29	0.05	0.90	0.67
*GM12878*	CAGT	N/A^a^	N/A	N/A	N/A	0.76	1.00
	Magnitude	N/A	N/A	N/A	N/A	0.82	1.00
	SOM-VN	0.60	0.50	N/A	N/A	0.82	0.97
*B-Cell (L)*	CAGT	N/A	N/A	0.12	0.18	0.81	0.76
	Magnitude	0.07	0.94	0.60	0.15	0.05	0.00
	SOM-VN	0.63	0.13	0.25	0.28	0.83	0.88
*B-Cell (H)*	CAGT	N/A	N/A	0.27	1.00	0.22	0.00
	Magnitude	0.06	0.99	0.11	0.00	0.14	0.00
	SOM-VN	0.58	0.36	0.43	0.88	0.93	0.72
*Brain (L)*	Magnitude	N/A	N/A	N/A	N/A	0.84	1.00
	SOM-VN	N/A	N/A	N/A	N/A	0.83	1.00
*Brain (H)*	Magnitude	0.12	1.00	N/A	N/A	N/A	N/A
	SOM-VN	0.50	0.33	0.37	0.32	0.81	0.89
*H1 (L)*	CAGT	0.05	0.71	N/A	N/A	0.64	0.10
	Magnitude	0.09	1.00	N/A	N/A	0.05	0.00
	SOM-VN	0.22	0.80	N/A	N/A	0.89	0.86
*H1 (H)*	CAGT	N/A	N/A	N/A	N/A	0.68	1.00
	Magnitude	N/A	N/A	N/A	N/A	0.78	1.00
	SOM-VN	0.57	0.57	N/A	N/A	0.76	0.96
*Heart (L)*	CAGT	N/A	N/A	0.42	0.05	0.76	0.99
	Magnitude	N/A	N/A	0.14	0.00	0.76	1.00
	SOM-VN	0.73	0.04	0.45	0.45	0.84	0.92
*Heart (H)*	CAGT	N/A	N/A	0.36	0.94	0.78	0.20
	Magnitude	N/A	N/A	0.02	0.00	0.72	1.00
	SOM-VN	0.25	0.01	0.43	0.96	0.96	0.52
*HeLa (L)*	CAGT	N/A	N/A	N/A	N/A	0.74	1.00
	Magnitude	N/A	N/A	N/A	N/A	0.78	1.00
	SOM-VN	0.41	0.66	0.30	0.00	0.84	0.92
*HeLa (H)*	CAGT	0.25	0.12	N/A	N/A	0.65	0.94
	Magnitude	N/A	N/A	N/A	N/A	0.76	1.00
	SOM-VN	0.32	0.73	0.35	0.04	0.81	0.76
*Stomach (L)*	CAGT	N/A	N/A	N/A	N/A	0.69	1.00
	Magnitude	N/A	N/A	0.05	0.00	0.74	1.00
	SOM-VN	0.60	0.22	0.41	0.11	0.77	0.97
*Stomach (H)*	CAGT	0.22	0.14	0.30	0.80	0.65	0.18
	Magnitude	0.14	0.00	N/A	N/A	0.70	1.00
	SOM-VN	0.61	0.31	0.38	0.88	0.91	0.62

^a^N/A values indicate that no shapes were learned for the corresponding RE in the corresponding sample

Because the *k*-means procedure used by CAGT was unable to converge on a set of shapes for brain tissue (H), we did not include that evaluation. We also did not include brain tissue (L) because neither SOM-VN nor CAGT learned any shapes associated with promoters or enhancers. Notably, CAGT did not learn any promoter shapes in GM12878, B-cells, H1 (H), heart tissue (H, L), HeLa (L), or stomach tissue (L), whereas SOM-VN learned promoter-associated shapes for all samples in this evaluation. Furthermore, CAGT did not learn any enhancer-associated shapes in GM12878, H1, HeLa, or stomach tissue (L), whereas SOM-VN learned enhancer-associated shapes in all cell types but H1 and GM12878. Finally, in samples where CAGT learned promoter or enhancer associated shapes, SOM-VN exhibited considerable improvement. SOM-VN improved A549 promoter precision by 0.05 and recall by 0.33, B-cell (L) enhancer precision by 0.13 and recall by 0.10, heart tissue (L) enhancer precision by 0.03 and recall by 0.40, HeLa (H) promoter precision by 0.17 and recall by 0.61, and stomach tissue (H) promoter precision by 0.39, promoter recall by 0.17, enhancer precision by 0.38, and enhancer recall by 0.08. Weak precision and recall were also improved in these samples, with the exception of a 0.07 drop in recall for heart tissue (L) and a 0.18 drop in recall for HeLa (H).

To verify that the associations found with REs were due to shape and not to magnitude alone, we also learned associations between REs and maximum signal within a region. We then used these associations to assign chromatin state to GM12878, B-cells, H1 (H), heart tissue, HeLa cells, and stomach tissue. We found that not only did shapes learned on SOM-VN yield markedly better precision and recall than magnitude alone for enhancers and promoters in the majority of cases, but that using magnitude alone did not result in promoter or enhancer associations being made for the majority of cell types (Table 2). This suggests that shapes provide better chromatin state assignments than magnitude alone.

#### Comparison to PEAS for enhancer state assignment

Although PEAS performs a task similar to SOM-VN, it is markedly different in the following ways: (1) PEAS assigns chromatin state to peaks only, rather than assigning chromatin states to the entire genome, and (2) PEAS distinguishes enhancers from other REs after removing promoters, rather than assigning chromatin states that distinguish between multiple types of REs. To compare SOM-VN to PEAS, we adapted SOM-VN to distinguish enhancers from non-enhancers after removing promoters. We evaluated the performance of the SOM-VN workflow on the GM12878 cell line in order to directly compare to the published results for PEAS [32]. In this evaluation, we used the ground truth chromatin state assignments provided by the authors of PEAS and removed promoters within this ground truth. To emulate the evaluation of peaks only, as done by PEAS, we restricted our evaluations of precision and recall to bins above a 5 RPKM threshold. We chose this height because it corresponded to a typical peak height in DNase-seq data in line with our previous work (data not shown). We found that SOM-VN had consistent precision and recall performance across chromosomes.

The precision-recall (PR) area under the curve (AUC) score approached the PR AUC scores reported in [32] with genomic features included and improved upon performance reported in [32] when using peak features alone (Table 3). These data indicated that SOM-VN can annotate enhancers with similar accuracy to PEAS if the evaluation of state is constrained by signal intensity.

**Table 3:** Precision-Recall AUC was comparable to PEAS results in GM12878

RPKM cutoff	SOM-VN (5 RPKM)	PEAS (all features)	PEAS (shape only)
*Enhancer PR AUC*	0.81	0.85	0.75

To determine whether thresholding by signal intensity also increased precision and recall in the general case (i.e. when associating regions with promoters, enhancers, and weak RE), we also evaluated precision and recall on only signal above 5 RPKM for the general case and compared the resulting precision and recall to that of the general case without this cutoff. We found that this cutoff increased B-cell (L), A549, H1 (L), GM12878, and stomach tissue (L) promoter precision by more than 0.10 and H1 (H) promoter recall by more than 0.10, but did not find any changes of this magnitude in enhancers, weak REs, or promoters of other samples (Supplementary Table 7 in Additional file 1). This result suggests that using a signal intensity cutoff improves PR AUC of enhancer assignment when promoters are removed.

## Discussion

SOM-VN is the first workflow that supports chromatin state assignment using chromatin accessibility signal shapes from segmented regions spanning entire chromosomes, allowing for a reduced number of assays and richness of signal information without reliance on traditional peak calling. The ability to isolate shapes with a range of signal intensities in imbalanced chromatin accessibility signal is made possible by adding variable neighborhood sizing to SOM. Our experiments showed that there exists a relationship between REs and chromatin accessibility signal shape that can be generalized across chromosomes and across some cell types. Characteristics specific to REs also emerged. For example, enhancer-associated shapes in high SPOT samples tended to have multiple summits, in comparison to promoter-associated shapes which tended to have one tall summit. Finally, while no other method has attempted to associate segmented chromatin accessibility signal shape with multiple types of REs, CAGT and PEAS are the most similar methods of which we are aware. We showed that SOM-VN improved precision and recall beyond CAGT [39] and performed similarly to PEAS when applied to the task for which PEAS was designed [32].

We note that performance can also be cell-type specific or influenced by data quality (as measured using SPOT score). For instance, in B-cells, brain tissue, heart tissue, and stomach tissue, SOM-VN learned more enhancer-associated shapes in high SPOT samples than in low SPOT samples and also exhibited better overall enhancer precision and recall. However, in H1 cells, no enhancer-associated shapes were learned for either the high SPOT sample or the low SPOT sample.

We note that SOM-VN has some limitations that could be improved upon in future iterations. First, we selected a 4 kb region size by evaluating the Davies–Bouldin clustering index [40] for a range of region sizes in high SPOT brain tissue (see Additional file 2) to measure the separability of clusters. We considered a range from 2 kb (the maximum size of most CTCF, H3K4me3, and H3K27ac markers [40]) to 32 kb (a region size capable of encompassing intergenic regions [41], stretch enhancers [1] and super-enhancers [42]). A systematic evaluation of Davies–Bouldin on other cell types is an area of future work, as is incorporating additional region sizes, such as those in [1, 43, 44]. Notably, because promoters and enhancers are often smaller than 2 kb, the evaluation of smaller regions could reveal new insights. Second, we set the parameters (such as association cutoff, grid size, learning rate, and scaling factor) manually using observations of shape variability in the output. A future direction is thus to optimize the parameters using a grid search or other optimization technique.

## Conclusions

Assignment of chromatin state is a challenge due to the limitations of experimental validation on a large scale and the number of assays needed to computationally assign states associated with REs using histone modifications. For this reason, assigning chromatin state using chromatin accessibility signal alone would provide a less resource-intensive option for researchers. To this end, we developed SOM-VN to learn shapes and associate them with REs (weak REs, promoters, enhancers). We found that learned shapes were non-random and consistent across chromosomes and that using these shapes to assign chromatin state could improve precision and recall over existing methods. Our work is thus an important step toward expanding the utility of chromatin accessibility signal shape alone to assign chromatin state associated with REs.

## Methods

### Chromatin accessibility data

We used publicly available chromatin accessibility data aligned to hg38 from 8 different cell types with variations in SPOT score. These included cell lines A549 (low SPOT score), H1 (low and high SPOT scores), HeLa (low and high SPOT scores), and GM12878 (low SPOT score), primary B-cells from a 21-year-old adult male (high SPOT score) and a 37-year-old adult male (low SPOT score), and the following tissue samples: brain tissue from 1 male embryo at 58 days gestation and 1 embryo at 56 days gestation (sex not collected, high SPOT score), brain tissue from 1 male embryo at 105 days gestation (low SPOT score), heart tissue from 1 embryo at 96 days gestation (sex not collected, high SPOT score), heart tissue from a 3-year-old male (low SPOT score), stomach tissue from a female embryo at 98 days gestation (low SPOT score), and stomach tissue from a female embryo at 108 days gestation (high SPOT score). The function *gosr binbam* (available from https://github.com/wresch/gosr) was used to compute RPKM signal and smooth the signal into 50 bp bins. We segmented this signal into 4 kb training regions.

### Chromatin state assignment

We used publicly available ChromHMM annotations in hg38 as ground truth. We downloaded annotations for the following Roadmap epigenome ID’s: E114 (A549), E081 (male fetal brain tissue), E003 (H1), E116 (GM12878), E083 (fetal heart tissue), E117 (HeLa), E032 (primary B-cells from peripheral blood), and E092 (fetal stomach tissue). These annotations were based on a 15-state ChromHMM model from the Roadmap Epigenomics Project (https://egg2.wustl.edu/roadmap/web_portal/chr_state_learning.html).

### Training SOM-VN

SOM-VN is a modified version of SOM, a type of single-layer neural network where nodes are laid out in a grid. Each segmented 4 kb region in the input is mapped to its best matching node using a similarity metric computed on node weights during training, and the nodes in its neighborhood are then updated to reflect the region’s shape [34]. We modified SOM to include variable neighborhood sizing, in which each node’s neighborhood size is dependent on a scaling factor *λ*, determined by the maximum weight of the node. Because each node’s weights reflect a shape, variable neighborhood sizing boosts the influence of shapes with high maxima to mitigate the imbalance of the input signal towards low chromatin accessibility. We used a mini-batch variant of SOM [45] in SOM-VN to balance the tradeoff between memory and runtime [46]. For the details of the SOM-VN training algorithm, please see Additional file 2.

### Merging learned shapes

Some of the shapes learned using SOM-VN may be shifted versions of one another. To mitigate this, we performed a merging procedure across all pairs of shapes learned, which made use of cross-correlation as described in [47] and applied in [21]. For the details of this process, please see Additional file 2.

### Associating shapes with chromatin states

We used an overlapping procedure to associate shapes with ChromHMM annotations. We first matched each training region to its best matching shape using cross-correlation, then computed the overlap between these matched regions and the ChromHMM annotations. In this manner, we obtained the distribution of bins across annotations for regions matched to each shape. Because the original ChromHMM annotations separated each RE type into categories by epigenetic state (e.g. poised and active promoters), which was not the focus of our work, we simplified the chromatin state space to include the following:

- *Promoter* Active, flanking active, bivalent, poised, and flanking bivalent transcription start sites
- *Enhancer* Genic enhancers, enhancers, and bivalent enhancers
- *Weak* Heterochromatin and quiescent regions

Additional details are available in Additional file 2.

### Associating magnitudes with chromatin states

To find associations between REs and the maximum magnitude of a region, we first computed the maximum RPKM signal of each 4 kb region in each chromosome. We then computed the overlap between these regions and the ChromHMM annotations to find associations. Additional steps were conducted in the same manner as when associating shape with REs and are described in Additional file 2.

### Assigning chromatin state to new regions

To assign chromatin state to new chromosomes or cell types not used to learn shape (i.e. the testing data), we segmented the testing data into 8 kb regions overlapping each other by 4 kb. In this manner, we evaluated containment of shape rather than exact match, thereby avoiding mis-assignment due to signal shift. We then computed an ambiguity metric on each region: the ratio of the cross-correlation between a region and its matching shape, and the cross-correlation between the region and its second-best matching shape. To consolidate overlap, we used a dynamic programming approach that minimized the sum of ambiguities. This assignment process resulted in a BED file containing the start and end points of each 8 kb region as well as the RE most associated with its chromatin state.

### Precision and recall

We used the true positive rate (TPR), false positive rate (FPR), true negative rate (TNR), and false negative rate (FNR) to evaluate the precision (TPR/(TPR + FPR)) and recall (TPR/(TPR + FNR)) on chromatin state assignments made using the SOM-VN workflow with respect to each RE. We defined a true positive for a given RE type *A* as a region which was both matched to *A* and for which its percentage of coverage in ChromHMM across all RE types was maximized in *A*. Likewise, we defined a false positive as a region that was matched to *A* but for which its percentage of coverage in ChromHMM across all RE types was not maximized in *A*, a true negative as a region that was not matched to *A* and for which its percentage of coverage in ChromHMM across all RE types was not maximized in *A*, and a false negative as a region that was not matched to *A* but for which its percentage of coverage in ChromHMM across all RE types was maximized in *A*.

### Null models

We included 2 types of null models in our analysis: the permuted signal model, which represented shapes learned randomly assuming no pattern of variations in the signal, and the permuted ChromHMM model, which represented a case where no true associations between shape and annotation existed. To build the permuted signal model, we permuted signal intensities in the 50 bp bins of the RPKM signal file before segmenting the signal into 4 kb regions and learning shapes using these permuted signal regions. We then associated these shapes with REs from ChromHMM annotations to obtain associations between random signal and chromatin state. To build the permuted ChromHMM model, we permuted the positions of ChromHMM annotations without modifying the annotation sizes.

## Supplementary Information

**Additional file 1.** Supplementary Tables. This file contains Supplementary Tables 1–7.

**Additional file 2.** Supplementary Figures and Methods. This file contains the supplementary figures referenced in the manuscript and details the methodology for segmenting regions, computing the Davies-Bouldin Index, training the SOM-VN, merging learned shapes, and associating learned shapes with RE.

## Data Availability

The datasets analyzed during the current study are available from ENCODE at the accession numbers ENCSR000ELW (https://www.encodeproject.org/experiments/ENCSR000ELW/), ENCSR595CSH (https://www.encodeproject.org/experiments/ENCSR595CSH/), ENCSR420RWU (https://www.encodeproject.org/experiments/ENCSR420RWU/), ENCSR000EMT (https://www.encodeproject.org/experiments/ENCSR000EMT/), ENCSR794OFW (https://www.encodeproject.org/experiments/ENCSR794OFW/), ENCSR000EJN (https://www.encodeproject.org/experiments/ENCSR000EJN/), ENCSR305UJX (https://www.encodeproject.org/experiments/ENCSR305UJX/), ENCSR911LTI (https://www.encodeproject.org/experiments/ENCSR911LTI/), ENCSR000EJT (https://www.encodeproject.org/experiments/ENCSR000EJT/), ENCSR959ZXU (https://www.encodeproject.org/experiments/ENCSR959ZXU/), ENCSR247IUJ (https://www.encodeproject.org/experiments/ENCSR247IUJ/), ENCSR381PXW (https://www.encodeproject.org/experiments/ENCSR381PXW/), ENCSR511GQA (https://www.encodeproject.org/experiments/ENCSR511GQA/), and ENCSR208DMX (https://www.encodeproject.org/experiments/ENCSR208DMX/) and from the Roadmap Epigenomics Project 15-state ChromHMM model results (https://egg2.wustl.edu/roadmap/data/byFileType/chromhmmSegmentations/ChmmModels/coreMarks/jointModel/final/E114_15_coreMarks_hg38lift_mnemonics.bed.gz, https://egg2.wustl.edu/roadmap/data/byFileType/chromhmmSegmentations/ChmmModels/coreMarks/jointModel/final/E081_15_coreMarks_hg38lift_mnemonics.bed.gz, https://egg2.wustl.edu/roadmap/data/byFileType/chromhmmSegmentations/ChmmModels/coreMarks/jointModel/final/E003_15_coreMarks_hg38lift_mnemonics.bed.gz, https://egg2.wustl.edu/roadmap/data/byFileType/chromhmmSegmentations/ChmmModels/coreMarks/jointModel/final/E116_15_coreMarks_hg38lift_mnemonics.bed.gz, https://egg2.wustl.edu/roadmap/data/byFileType/chromhmmSegmentations/ChmmModels/coreMarks/jointModel/final/E117_15_coreMarks_hg38lift_mnemonics.bed.gz, https://egg2.wustl.edu/roadmap/data/byFileType/chromhmmSegmentations/ChmmModels/coreMarks/jointModel/final/E083_15_coreMarks_hg38lift_mnemonics.bed.gz, https://egg2.wustl.edu/roadmap/data/byFileType/chromhmmSegmentations/ChmmModels/coreMarks/jointModel/final/E092_15_coreMarks_hg38lift_mnemonics.bed.gz, and https://egg2.wustl.edu/roadmap/data/byFileType/chromhmmSegmentations/ChmmModels/coreMarks/jointModel/final/E032_15_coreMarks_hg38lift_mnemonics.bed.gz). GM12878 chromatin state assignments were obtained directly from the authors of PEAS. Our code is available from https://github.com/taraeicher/SOM_VN. Scripts are written in Python and Bash for the Unix environment.

## Abbreviations

A549: Carcinomic human alveolar basal epithelial cell line
ATAC-seq: Assay for transposase-accessible chromatin with high-throughput sequencing
bp: Base pairs
BAM: Binary alignment map
BED: Browser extensible data
CAGT: Clustering aggregation tool
ChIP-seq: Chromatin immunoprecipitation followed by sequencing
CoSBI: Coherent and shifted bicluster identification
CpG: 5′—C—phosphate—G—3'
CTCF: CCCTC-binding factor
DNase-seq: Deoxyribonuclease I hypersensitive sites sequencing
ENCODE: Encyclopedia of DNA elements
FAIRE-seq: Formaldehyde-assisted isolation of regulatory elements
FNR: False negative rate
FPR: False positive rate
GC: Guanine-cytosine
GM12878: Human B-lymphoblastoid cell line
H1: H1 human embryonic stem cell line
H3K27ac: Acetylation of lysine 27 on histone H3 protein subunit
H3K4me3: Trimethylation of lysine 4 on histone H3 protein subunit
HeLa: Human epithelial carcinoma cell line
hg38: Homo sapiens (human) genome assembly GRCh38
kb: Kilobase pairs
MNase-seq: Micrococcal nuclease digestion with deep sequencing
NiCE-seq: Nicking enzyme assisted sequencing
NOMe-seq: Nucleosome occupancy and methylome sequencing
PEAS: Predicting enhancers from ATAC-Seq data
PR AUC: Precision-recall area under the curve
RE: Regulatory element
RPKM: Reads per kilobase of transcript, per million
SOM: Self-Organizing Map
SOM-VN: Self-Organizing Map with Variable Neighborhoods
SPOT: Signal portion of tags
TNR: True negative rate
TPR: True positive rate
TSS: Transcription start site(s)
WIG: Wiggle file format
