# Supplementary material for: Self-organizing maps with variable neighborhoods facilitate learning of chromatin accessibility signal shapes associated with regulatory elements
Source: BMC Bioinformatics. 2021 Jan 30;22:35. doi: 10.1186/s12859-021-03976-1 (PMC7847148; doi:10.1186/s12859-021-03976-1)
Supplement: Supplementary file 2 — Additional file 2. Supplementary Figures and Methods. This file contains the supplementary figures referenced in the manuscript and details the methodology for segmenting regions, computing the Davies-Bouldin Index, training the SOM-VN, merging learned shapes, and associating learned shapes with RE. [file 12859_2021_3976_MOESM2_ESM.docx]

Supplementary Figures and Methods

**
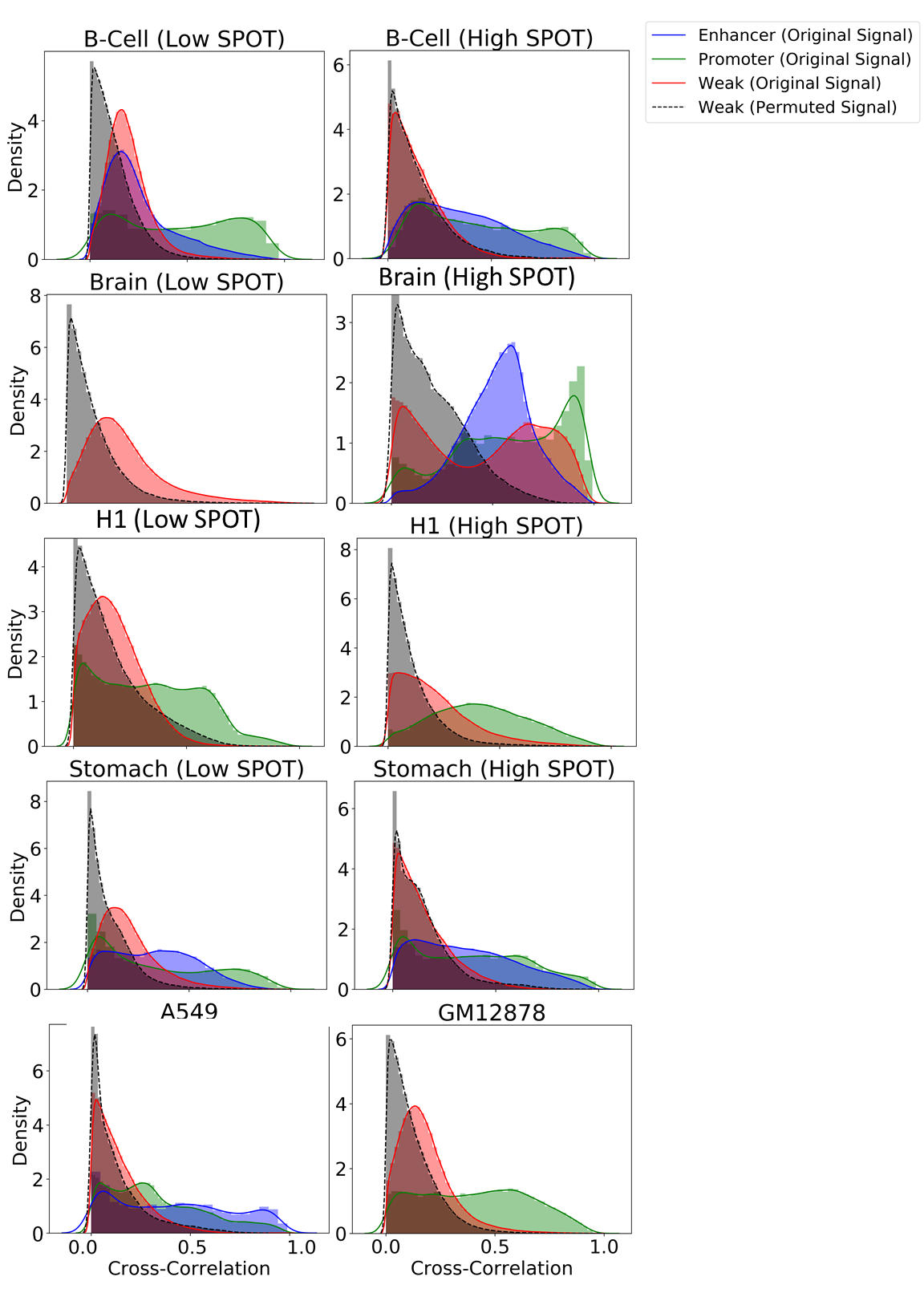
**

**Supplementary Figure 1. Extension of Figure 2 from the main text.** The density plots indicate the distribution of cross-correlations between regions and their matching shapes for all regions, and the color denotes the RE with which the matching shape is associated. Statistically higher cross-correlations were found between shapes learned using the original, unpermuted signal and their matching regions than between shapes learned using permuted signal and their matching regions.

# Segmenting 4 kb Regions

To avoid peaks being split between regions during segmentation, we first segmented 6 kb regions and then selected a smaller 4 kb sub-region within each using an optimization criterion of our design. We allowed for overlap of 1 kb between 6 kb regions, effectively restricting the center of the 4 kb region to be no more than 1 kb from either edge of the 6 kb region (Supplementary Figure 2a).

Our optimization criterion was designed to select sub-regions such that the shapes within them would be centered. We used signal distance from the center of the selected region as a proxy for centeredness of the shape and measured this using a linearly weighted sum of signals, with the highest weight at the center and negative weights in the outer ¼ of the region’s bins. We then applied our criterion to a sliding window of 4 kb across each 6 kb region to find the optimal sub-region.

This criterion is defined in Supplementary Equation 1 and illustrated in Supplementary Figure 2b, where *d* is the number 50 bp of bins in the 4 kb sub-region, i.e. 800, *m* is the number of 50 bp bins in the 1 kb margin, i.e. 100, *x* represents the signal intensities of all 50 bp bins across the 4 kb region, and *c* represents the rate of weight decrease with respect to distance from the center of the sub-region. Crucially, we note that this equation was applied only for choosing which 4 kb sub-region to select for training; the signal intensities of the regions themselves were not altered before SOM-VN training.

$\max_{0\leq i\leq2m} \sum_{j=1}^{d+i} x_{j}*\frac{\left( cd \right)-\left| j-\frac{d}{2} \right|}{cd}$ (Supplementary Equation 1)

**Supplementary Figure 2. Selection of 4 kb training regions.** (A) We selected 6 kb regions with 1 kb overlap before choosing the final 4 kb training regions, (B) We selected the training regions by computing a weighted sum of signal intensities for all possible 4 kb sub-regions within the 6 kb region and choosing the 4 kb sub-region with the maximum sum according to Supplementary Equation 1.

# Davies-Bouldin Index

To evaluate shape separability, we evaluated the Davies-Bouldin clustering index on region sizes ranging from 2 kb to 32 kb for a subset of chromosomes in brain tissue. The Davies-Bouldin index measures the ratio of intra-cluster similarity to the inter-cluster similarity; in an optimal case, it should be near 0. The index is defined in Supplementary Equation 2, where *C_i_* is the centroid of cluster *i*, *N_i_* is the size of the training data associated with *i*, *N* is the total size of the training data, and *X_k_* the signal from a single region. In our application, each shape corresponded to its own cluster. The shape itself represented the centroid, and all regions annotated with that shape formed the rest of the cluster. We then learned shapes using SOM-VN for each segmentation size.

$DB= \frac{1}{N}\sum_{i=1}^{N} \max_{j\neq i} \frac{\sqrt{\frac{1}{N_{i}}\sum_{k=1}^{N_{i}} \left\| X_{k}-C_{i} \right\|}+\sqrt{\frac{1}{N_{j}}\sum_{k=1}^{N_{j}} \left\| X_{k}-C_{j} \right\|}}{\left\| c_{i}-c_{j} \right\|}\frac{1}{N}$ (Supplementary Equation 2)

For most of the chromosomes evaluated in this experiment, 4 kb training regions show a lower (better) Davies-Bouldin index than other training region sizes, indicating that a training region size of 4 kb results in a set of shapes that captures the range of data better than other region sizes.


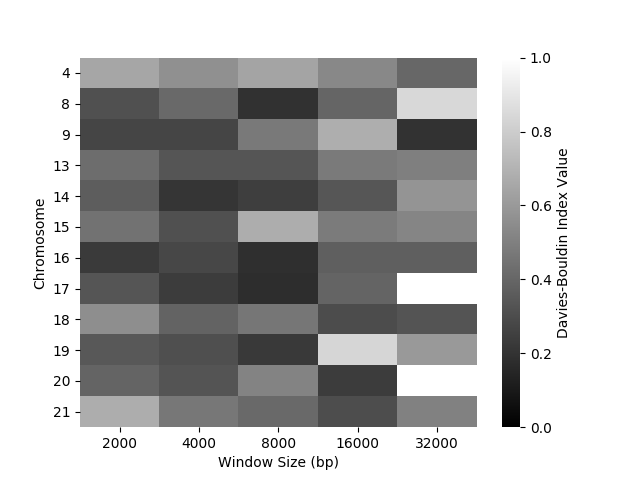


**Supplementary Figure 3.  Davies-Bouldin index across brain tissue chromosomes.** Across the chromosomes listed, 4 kb exhibited the most consistently low index (where 0 is optimal). Other region sizes exhibited low indices for some chromosomes, but these were not broadly applicable. Here, “Window Size” indicates the size of the training region.

# Details of SOM-VN Algorithm

The training procedure for SOM-VN is described in Supplementary Algorithm 1 below, where:

- *w* is the set of weights over all nodes in the grid.
- *λ* is the neighborhood scaling factor
- *t* is the current training epoch.
- *ι* is the total number of epochs set for the algorithm.
- *m* is the size of the minibatch used for training (i.e. the number of regions fed into the algorithm at once).
- *n* is the total number of training regions.
- *α_t_* is the learning rate for the algorithm at epoch *t*, which determines how much each node’s weights should change with respect to its matching regions
- *σ_t_* is the neighborhood size at epoch *t*, which influences how much a node’s weights change as a function of its distance from the BMU
- *Θ(w_i_, w_b_,t)* is the neighborhood function computing the update to be applied to *w_i_* given the regions mapping to *w_b_* at epoch *t.*
- *μ* is the index of the current minibatch within the current epoch.
- *g* is the total number of nodes in the grid, which affects the total number of shapes that can be learned*.*

PROCEDURE Train_SOM_VN:

w ← RAND(UNIF(1, 100)), t ← 0, μ ← 0

WHILE t < ι:

α_t_ ← Supplementary Equation 3

σ_t_ ← Supplementary Equation 4

WHILE μ < n / m: ◄Train using minibatches.

w_b,t_ ← Supplementary Equation 5 ◄Find set of BMU’s.

Θ(w_i,t_, w_b,t_, σ_t_) ← Supplementary Equation 6 ◄Update neighborhood function.

i ← 0

WHILE i < g: ◄Update the weight of all nodes in the grid.

w_i,t_ ← Supplementary Equation 7

i ← i + 1

μ ← μ + 1

t ← t + 1

**Supplementary Algorithm 1: Procedure for training SOM-VN.** The training procedure is an iterative process. Comments are indicated above by block arrows and assignment by arrows. Equations used in the algorithm are referenced by number.

$\alpha_{t}=\alpha_{0}*\left( 1-\frac{t}{\iota} \right)$ (Supplementary Equation 3)

$\sigma_{t}=\sigma_{0}*\left( 1-\frac{t}{\iota} \right)$ (Supplementary Equation 4)

$w_{b,t}\left( x_{j} \right)=\min_{i} d\left( w_{i,t}, x_{j} \right)$ (Supplementary Equation 5)

$\theta\left( w_{i,t}, w_{b,t},\sigma_{t} \right)= e^{-\frac{\sqrt{\left( Coord(i)- Coord(b) \right)^{2}}}{{\sigma_{t}}^{2}\lambda}}$ (Supplementary Equation 6)

$w_{i,t}=\alpha_{t}w_{i,t-1}+\left( 1-\alpha_{t} \right)*\frac{\sum_{j=0}^{m} \theta\left( w_{i,t}, w_{b,t},\sigma_{t} \right)*x_{j}}{\sum_{j=0}^{m} \theta\left( w_{i,t}, w_{b,t},\sigma_{t} \right)}$ (Supplementary Equation 7)

# Merging Shapes Using Cross-Correlation

We compute the maximum cross-correlation (defined in Supplementary Equation 8) of two shapes with shifts up to 1 kb, or ¼ the size of the region. We choose this cutoff to ensure that at least ¾ of the signal intensities comprising the shape are involved in computing the cross-correlation; other shifts could also be considered. Cross-correlation, a normalized cosine similarity metric used in signal processing, is defined below, where *w_i_* is the set of weights for node *i,* *d* is the dimensionality of each shape (i.e. 80), and *k* is the shift.

$CC\left( w_{i},w_{j} \right)=\frac{w_{i}\left[ 0\ldots k \right]*w_{j}\left[ d-k\ldots d \right]-\frac{\sum_{l=0}^{k} w_{i}\left[ l \right]*\sum_{l=d-k}^{d} w_{j}\left[ l \right]}{k}}{\sqrt{\left( \sum_{l=0}^{k} {w_{i}\left[ l \right]}^{2}-\frac{\left( \sum_{l=0}^{k} w_{i}\left[ l \right] \right)^{2}}{k} \right)*\left( \sum_{l=d-k}^{d} {w_{j}\left[ l \right]}^{2}-\frac{\left( \sum_{l=d-k}^{d} w_{j}\left[ l \right] \right)^{2}}{k} \right)}}$ (Supplementary Eq. 8)

Cross-correlation thus detects similarity between two shapes even if they differ in phase. Notably, we restricted the range of *k* to include the maximum signal of the shape in the calculation, and we restricted evaluation of cross-correlation to pairs in which one shape has no more than twice the maximum signal intensity of the other. This restriction ensures that signal intensity is also included in the characterization of shape. We merged shapes with maximum cross-correlation values above 0.75; this cutoff was also used in the ChIP-seq-based RE annotation method CoSBI to construct bi-clusters of ChIP-seq peaks (25).

We note that the merging process described above requires a pairwise comparison of all shapes learned by the SOM-VN grid. If the grid is large, it is possible to reduce the computational intensity of this step by running *k*-means clustering on the SOM-VN output before the merging process.

# Cutoffs for Associating Shapes with RE Annotations

For each learned shape, we calculated the percentage of contained regions annotated as promoters, enhancers, weak, or other. To attribute a RE annotation *A* to a given shape, we required at least 50% of all regions within a shape to be labeled as *A* and at most half of those regions labeled as *A* to be labeled any of the other RE annotations. These shapes were saved in a RE annotation association file with their RE annotation association. Notably, shapes that did not meet this criterion were not associated with any RE annotations and subsequently were not used in annotating new regions. These shapes were also included in the RE annotation association file, with the association listed as Unknown.
